# Characterization of oscillatory changes in hippocampus and amygdala after deep brain stimulation of the infralimbic prefrontal cortex

**DOI:** 10.14814/phy2.12854

**Published:** 2016-07-22

**Authors:** Ana Cervera‐Ferri, Vicent Teruel‐Martí, Moises Barceló‐Molina, Joana Martínez‐Ricós, Aina Luque‐García, Sergio Martínez‐Bellver, Albert Adell

**Affiliations:** ^1^ Neuronal Circuits LaboratoryDepartment of Human Anatomy and EmbriologyFaculty of Medicine and OdontologyUniversity of ValenciaValencia46010Spain; ^2^ Instituto de Investigación Sanitaria La FeValencia46026Spain; ^3^ Department of Cell Biology and ParasitologyFaculty of Medicine and OdontologyUniversity of ValenciaValencia46010Spain; ^4^ Institute of Biomedicine and Biotechnology of CantabriaIBBTEC (CSIC University of Cantabria)Santander39011Spain

**Keywords:** Antidepressant, brain oscillations, local field potential, modulatory index, mutual information

## Abstract

Deep brain stimulation (DBS) is a new investigational therapy that has generated positive results in refractory depression. Although the neurochemical and behavioral effects of DBS have been examined, less attention has been paid to the influence of DBS on the network dynamics between different brain areas, which could contribute to its therapeutic effects. Herein, we set out to identify the effects of 1 h DBS in the infralimbic cortex (IL) on the oscillatory network dynamics between hippocampus and basolateral amygdala (BLA), two regions implicated in depression and its treatment. Urethane‐anesthetized rats with bilaterally implanted electrodes in the IL were exposed to 1 h constant stimulation of 130 Hz of frequency, 60 *μ*A of constant current intensity and biphasic pulse width of 80 *μ*sec. After a period of baseline recording, local field potentials (LFP) were recorded with formvar‐insulated stainless steel electrodes. DBS of the IL increased the power of slow wave (SW, <1.5 Hz) and theta (3–12 Hz) frequencies in the hippocampus and BLA. Furthermore, IL DBS caused a precise coupling in different frequency bands between both brain structures. The increases in SW band synchronization in hippocampus and BLA after DBS suggest that these changes may be important for the improvement of depressive behavior. In addition, the augmentation in theta synchrony might contribute to improvement in emotional and cognitive processes.

## Introduction

Major depressive disorder (MDD) is characterized by a group of symptoms, which are associated mainly –but not solely – to malfunction of several brain regions such as prefrontal cortex (PFC), hippocampus, and amygdala. Based on positron emission tomography (PET) studies, Mayberg (1997) proposed a model of depression delineated by a hypoactive dorsal compartment and a hyperactive ventral compartment in the brain. This view received further support in subsequent work (Mayberg et al. 1999; Drevets 2000). This model also contemplates that an effective response to antidepressant treatment would normalize these altered activities in both components. Functional magnetic resonance imaging (fMRI) studies also revealed that the amygdala is overactive in depression and responds excessively to negative stimuli (Sheline et al. 2001; Siegle et al. 2002), whereas the hippocampal formation is generally hypoactive in MDD (Seminowicz et al. 2004). This reduced activity can further result in a diminished hippocampal volume (Bremner et al. 2000; MacQueen and Frodl 2011). Overall, the amygdala and hippocampus interact cooperatively to mediate emotion‐related behaviors such as fear responses (LeDoux 2000; Phelps 2004). In addition to hippocampus and amygdala, the PFC plays a crucial role in the circuitry involved in the regulation of emotional processes and, consequently, impairment of the PFC‐hippocampus‐amygdala is one of the most important features of MDD (Hastings et al. 2004).

Deep brain stimulation (DBS) is a new investigational therapy that has generated positive results in refractory depression. Initial clinical studies demonstrated that overactivity of the subgenual cingulate gyrus (SCG) in depression normalized after DBS, together with relief of symptoms (Mayberg et al. 2005). However, this early enthusiasm has not been confirmed by a recent controlled and randomized trial (Dougherty et al. 2015). In the rodent brain, the infralimbic (IL) region of the medial PFC (mPFC) and the ventral part of the prelimbic mPFC, as a whole, is considered to be homologous to the SCG (Gabbott et al. 2003; Uylings et al. 2003). This is further supported for the similar connectivity of IL and SCG with other brain regions implicated in the pathophysiology of depression, such as hippocampus and amygdala (Heidbreder and Groenewegen 2003). Furthermore, DBS of the IL has been shown to have antidepressant‐like and anxiolytic‐like effects in the forced swim and the novelty‐suppressed feeding tests (Hamani et al. 2010; Jiménez‐Sánchez et al. 2016). In previous work, we have demonstrated that 1 h DBS of the IL increases the release of glutamate, serotonin (5‐hydroxytryptamine, 5‐HT), dopamine, and noradrenaline in the mPFC, which might contribute to the antidepressant‐like activity (Jiménez‐Sánchez et al. 2016). Although the neurochemical and behavioral effects of DBS have been examined, less attention has been paid to the influence of DBS on the network dynamics between different brain areas, in which an increased synchrony in oscillatory activity is assumed to support neural communication (Fell and Axmacher 2011). In this regard, it has been postulated that the effects of DBS may be partly related to synchronization and coherence of oscillatory activity in brain structures implicated in the circuitry of mood disorders (Ewing and Grace 2013). Neumann et al. (2014) provided the first evidence that oscillations in the 8–14 Hz band in the SCG may be suggestive of negative mood in MDD. Furthermore, it has been recently proposed that the suppression of frontal gamma oscillations and increased theta‐gamma coupling through DBS is likely mediated by both SCG activation of inhibitory circuits and an enhancement of plasticity in the frontal cortex and that the activation of both pathways may explain the therapeutic properties of DBS (Sun et al. 2015). Recent studies using EEG analysis reported that depressed people exhibited reductions of slow wave sleep (Duncan et al. 2013) and that ketamine and DBS increased slow wave (SW) and theta activity, respectively (Broadway et al. 2012; Duncan et al. 2013). Finally, it has been further proposed that intrinsic local beta oscillations in the SCG was related to depressive symptoms in treatment‐resistant depression (Clark et al. 2016).

To gain a better knowledge of the network effects of DBS, in the present work, we set out to examine in the urethane‐anesthetized rat the effects of 1 h IL DBS on the oscillatory network dynamics between hippocampus and basolateral amygdala (BLA), in the understanding that these changes could be associated with improvement of cognitive processes and reduction in anxious behavior seen in depressive‐like states.

## Materials and Methods

### Animals and surgery

A total number of six male Wistar rats (Janvier Labs; Le Genest‐Saint‐Isle, France) weighing 300–400 g were used. The animals were kept under controlled temperature of 22 ± 2°C and a 12 h lighting cycle. Food and water were always freely available. All experimental procedures were approved by the University of Valencia Animal Care and Use Committee in strict compliance with the Spanish (R.D. 53/2013) and European (Directive 2010/63/EU of the European Parliament and of the Council, 22 September 2010, on the protection of animals used for scientific purposes) legislation.

Rats were anesthetized with an intraperitoneal injection of 1.5 g/kg of urethane, placed on a stereotaxic frame (David Kopf Instruments, Tujunga, CA) and their body temperature kept at 37°C with a heating pad. The surgery area was infiltrated with 5% lidocaine. Level of anesthesia was maintained as required by supplemental doses of urethane (20% of initial dose). Trephine holes were drilled in the skull and recording electrodes placed at the dorsal hippocampus (A: −2.5 mm, L: 2 mm, and H: −3 mm) and BLA (A: −2.2 mm, L: 5 mm, and H: −8.7 mm), both in the left hemisphere. A stainless steel screw was implanted in the occipital bone as reference. Stimulating electrodes were implanted bilaterally into the infralimbic region of the mPFC (A: 3.2 mm, L: 0 mm, and H: −5.2 mm). All coordinates were taken from bregma and the surface of the skull according to Paxinos and Watson (2005).

### Recording and stimulation parameters

Local field potentials (LFP) were recorded with formvar‐insulated stainless steel monopolar macroelectrodes of 120 *μ*m diameter (World Precision Instruments, Sarasota, FL). Two electrodes were twisted allowing 1 mm between both intracerebral poles for the stimulation. Signals were recorded with a first amplification (×100, model p511 AC Grass Preamplifier) followed by a second amplification (×10, MPLI 4G21; Cibertec, Madrid, Spain), filtered in the 0.1–500 Hz band and sampled at 1 kHz (MK‐1041; Cambridge Electronics Design, Cambridge, UK).

After 900 sec of baseline recording, rats were subjected to 1 h DBS with the following parameters, which had demonstrated clinical relevance in the treatment of depression, that is, 130 Hz of frequency, 60 *μ*A of constant current intensity and biphasic pulse width of 80 *μ*sec. These parameters were chosen for being clinically relevant, but the intensity of stimulation was adapted to impact on a smaller volume of tissue in the rat (Srejic et al. 2015). This DBS protocol produced no visible lesions in the mPFC under microscopy with cresyl violet staining.

### Data analysis

All the analyses were performed off‐line in the Matlab development environment (The MathWorks, Natick, MA) using self‐developed and built‐in routines. First, LFPs were down‐sampled to 200 Hz and filtered between 0.1 and 100 Hz. The resultant signals were digitally notch filtered with a Butterworth bandstop filter around 50 ± 3 Hz, applied in both the forward and reverse directions to remove phase distortions. All the signals were z‐score normalized.

#### Spectral analysis

All the changes were assessed by comparison with initial baseline period. LFPs were analyzed in the frequency domain through standard power spectral analysis, using a nonparametric approach based on the Fast Fourier Transform. We calculated the power spectrum of the raw signal by means of the Welch's averaged method with Hanning windowing. The signal was divided into segments composed of 2048 samples without overlap, with a frequency resolution of 0.24 Hz. The analysis focused on five bands of interest: SW oscillations (<1.5 Hz), delta (1.5–3 Hz), theta (3–12 Hz), spindles (12–16 Hz), beta (16–30 Hz), and gamma (30–100 Hz). In addition, the total spectral power (0.1–100 Hz) was considered.

#### Time‐frequency analysis

To reveal oscillatory activity and their temporal changes in the LFP time‐series, the continuous wavelet transform was computed using the algorithm of Torrence and Compo (1998). Briefly, the signal was convoluted with complex Morlet's wavelet:

$$\psi_{0}\left(s\right)=\pi^{-1/4}e^{i\omega_{0}s}e^{-s^{2}/2}$$

With *s* as the scale (1/period) and a central frequency ω_*0*_ *=* 2π* *Hz, providing a good balance between time and frequency resolution (Farge 1992). This transformation is well suited to describe phasic or transient events by multiscale decomposition in the time and frequency domains. The wavelet transform output at each time, *W*(*s*), leads to power ($|W\left(s\right)|$) and phase $\left(\tan^{-1}Re\left\{W\left(s\right)\right\}/Im\left\{W\left(s\right)\right\}\right)$of the signal at each scale *s* (frequency). Power values were normalized to scale, $s^{-1}{\left|W(s)\right|}^{2}$, to avoid scale ‐dependent biased values (Liu et al. 2007).

The correlation between pairs of signals was first studied using wavelet cross‐spectrum, defined as *W*_*XY*_(*s*) = *W*_*X*_(*s*) * *W*_*Y*_(*s*), that is, the complex conjugation of the wavelet transforms of the two time series X and Y, in the wavelet scale domain (*s*). Thus, cross‐wavelet power can be defined as$\left|W_{XY}\left(s\right)\right|$, and reveals time‐frequency regions with high common power. Furthermore, the wavelet coherence can be defined as the square of the cross‐spectrum normalized by the individual power spectra in the range 0–1. Phase‐locking values (PLV) were defined as a measure of the stationarity of the phase differences in a temporal window and therefore of the phase‐depending synchrony (Lachaux et al. 1999).

#### Phase‐amplitude coupling

Cross‐frequency interactions between different frequency bands of a signal were assessed by the modulation index (MI), described by Tort and colleagues (Tort et al. 2010). In brief, raw data are filtered in the two frequency ranges of interest. The instantaneous phase of the slow wave and the amplitude of the fast oscillation are then both computed by means of the Hilbert transform. In our case, the MI value was determined to test the amplitude locking of all frequencies in the range 1.5–100 Hz to the SW phase. We extracted the phase of the single filtered SW trace with low‐pass filter below 1.5 Hz and converted to the range −*π* and +*π*, with wave trough as the 0 radians. The amplitude of the fast range of frequencies was calculated by taking the filtered trace using the absolute value of the Hilbert‐transformed signal. The MI is a normalized measure that reflects how well the instantaneous amplitude of a faster oscillation is phase‐locked to the underlying SW cycle. Therefore, the MI was calculated between SW phase and each 5 Hz frequency band spanning 1.5–100 Hz. In the case of phase‐amplitude coupling between both filtered signals, the amplitude distribution over the phase bins is nonuniform. Following Tort's calculation, MI is defined as a measure that quantifies the deviation from the uniform distribution with an adaptation of the Kullback–Leibler distance. The MI was considered statistically significant when its value was >2 × SD of the mean surrogate MI, constructed by 200 random permutations of the amplitude distribution.

#### Mutual information

Within the named information theory, mutual information is based in a mathematical calculation that provides a measure of the amount of information that is transferred from one random variable (one brain area) to another (another brain area). Based in the time‐frequency domain, power matrices were windowed into segments of 1 sec for frequencies in the 0.1–1.5 Hz range. Distribution of wavelet power values for each window was studied using the formalism of Shannon information theory (Shannon 1948). This approach shows sustained functional connectivity in the range of delimited frequencies between recorded regions.

### Statistics

Data are expressed as mean ± standard error of the mean (SEM). Statistical comparisons were made using nonparametric tests after confirming lack of normality (Shapiro–Wilks test) and homogeneity of variances (Levene test).

All the comparisons between baseline and the DBS effect were made using the Wilcoxon test. The threshold for significance was set at *P *<* *0.05. All analyses were performed using the SPSS 12.0.1 software package (SPSS Inc., Chicago, IL).

## Results

### Baseline conditions

**Figure 1:**
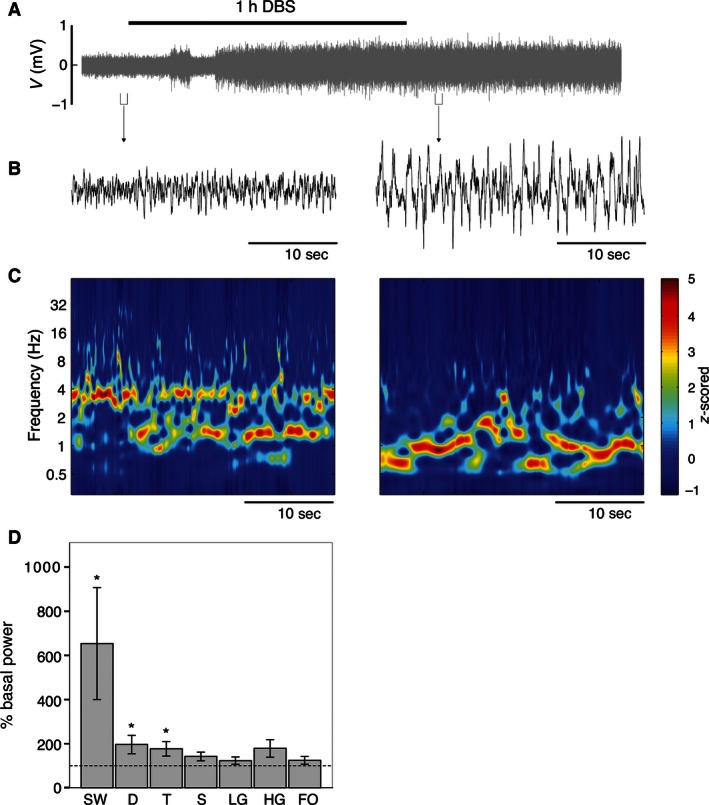
DBS activation of SW state in the hippocampus. (A) Raw LFP recorded in the CA1 area of the hippocampus, with detailed fragments represented in (B). Note the increase in amplitude minutes after the initiation of DBS. (C) Wavelet time‐frequency spectrograms showing the temporal structure of the predominant bands of the epochs depicted in (B), which demonstrates a switch to SW after DBS. (D) Statistical data showing the changes in the SW, delta, and theta frequency bands, with regard to pre‐DBS basal epoch (mean ± SEM, *P <* *0.05, Wilcoxon test). SW, slow oscillations; D, delta band; T, theta band; S, spindle band; LG, low‐gamma band; HG, high‐gamma band; FO, fast oscillations; DBS, deep brain stimulation.

Before the beginning of the DBS, the hippocampal LFP cycled between predominant delta periods and short spontaneous theta epochs, which depends on the level of anesthesia (Fig. 1A). In the basal situation (Figs. 1B, Left and Fig. S1), hippocampal activity consisted mostly of slow (0.5–1.5 Hz) and delta (1.5–3 Hz) waves: 31.8 ± 7.0% and 31.5 ± 4.6%, respectively, calculated as the mean of the power spectra in 30 10‐s windows. Theta oscillations (3–12 Hz) constituted 27.8 ± 5.1% of the basal recording. Higher frequency bands were also present with much lower proportion: 2.3 ± 0.4% of hippocampal spindles (12–16 Hz), 3.3 ± 0.8% of beta (16–30 Hz), 3.28 ± 0.79% of slow gamma (30–60 Hz), and 2.5 ± 0.6% of fast gamma (60–100 Hz) bands.

**Figure 2:**
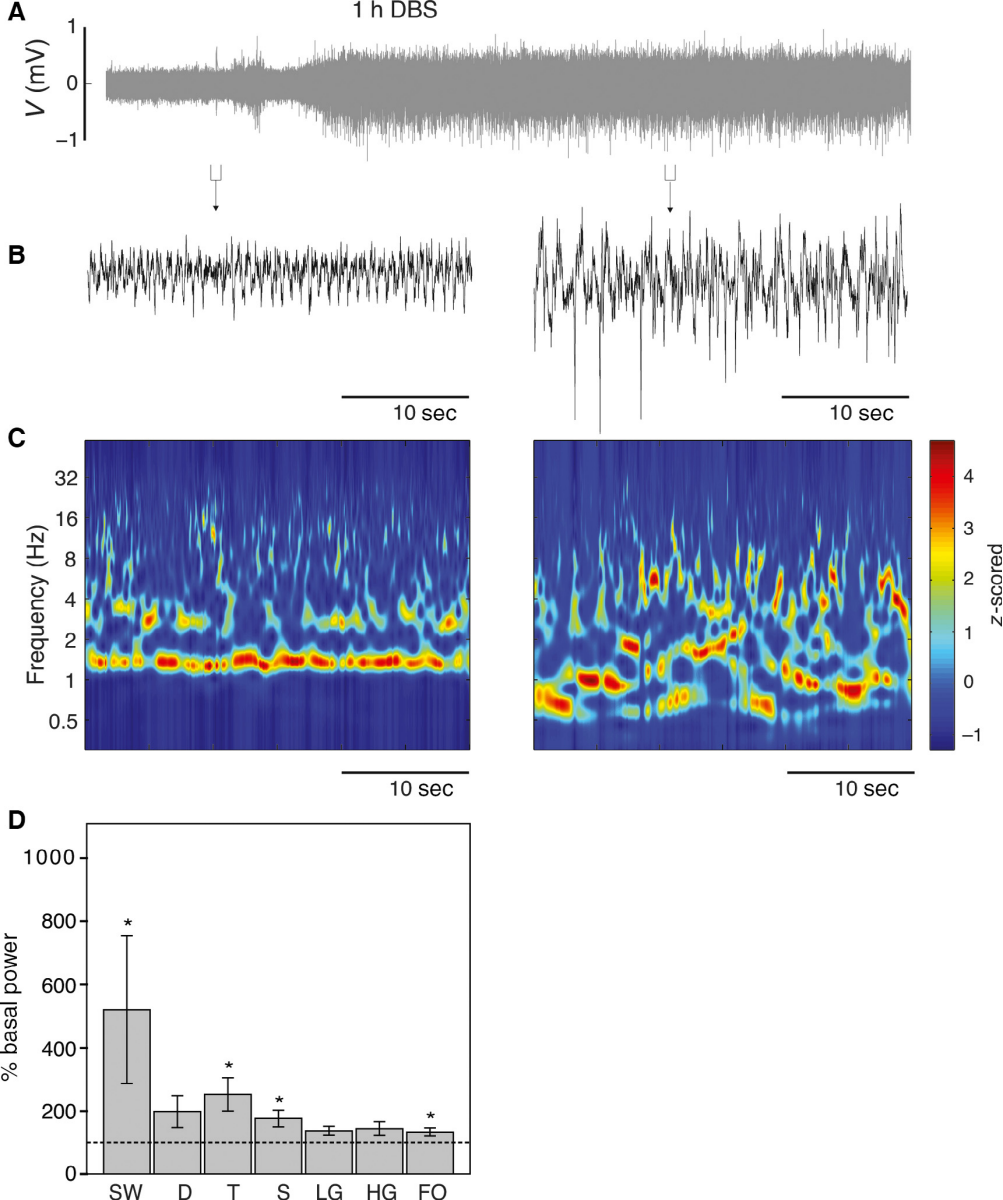
DBS activation of SW state in the BLA. (A) Raw LFP recorded in the BLA, with detailed fragments represented in (B). Similar to the to the hippocampal LFP profile, the amplitude of the oscillatory pattern increased after DBS. (C) Wavelet time‐frequency spectrograms show a SW state shared with higher frequency components. (D) Statistical data showing the changes in the SW, theta, beta, and fast‐oscillation frequency bands, with regard to pre‐DBS basal epoch (mean ± SEM, *P <* *0.05, Wilcoxon test). SW, slow oscillations; D, delta band; T, theta band; S, spindle band; LG, low‐gamma band; HG, high‐gamma band; FO, fast oscillations; DBS, deep brain stimulation.

The LFP activity of BLA (Fig. 2A) was very similar to that of hippocampus in basal, pre‐DBS conditions (Figs. 2B, left and Fig. S2), with 30.1 ± 5.3% of SW, 34.2 ± 3.7% of delta, 25.5 ± 1.8% of theta, 3.1 ± 0.7% of spindles, 3.7 ± 0.9% of beta, 0.9 ± 0.2% of slow gamma, and 2.6 ± 0.7% of fast gamma bands, expressed as percentage of mean power spectra. The hippocampus and BLA oscillatory patterns under basal conditions were also evident in color‐coded spectrograms (Figs. 1C, left and 2C, left, respectively).

### Effects of DBS on oscillatory profiles

1 h of DBS augmented LFP amplitude both in hippocampus (Figs. 1A and B, Right) and BLA (Figs. 2A and B, right). This was well evidenced by the power spectra (Figs. S1 and S2) and the power distribution of the wavelet spectrograms (Figs. 1C, right and 2C, right). The duration of this effect exceeded the extent of the recording period (>45 min after cessation of DBS).

To better delineate the variation in the oscillations, we analyzed the effects in the 0.4–80 Hz bands. Statistical differences were found in different bands for each channel when measuring the mean band power in 30 10‐sec windows during the effect condition. The percentage of basal power was calculated for each brain region. In the hippocampus (Fig. 1D), significant increases (Wilcoxon test) expressed as percentage from baseline were detected in SW (652.2 ± 252.3%; *P *<* *0.05), delta (196.4 ± 41.6%; *P *<* *0.05), and theta (176.9 ± 33.3%; *P *<* *0.05) bands after DBS. No significant changes were detected in beta, slow, and fast gamma bands (Fig. 1D). In BLA (Fig. 2D), DBS evoked significant increases with respect to basal power in SW (524.87 ± 237.97%; *P *<* *0.05), theta (252.78 ± 52.82%; *P *<* *0.05), spindles (175.82 ± 26.15%; *P *<* *0.05), and fast gamma (133.26 ± 13.22%; *P *<* *0.05) oscillations. Only a nonsignificant tendency was observed in the beta band (136.55 ± 14.63%; *P *=* *0.075).

**Figure 3:**
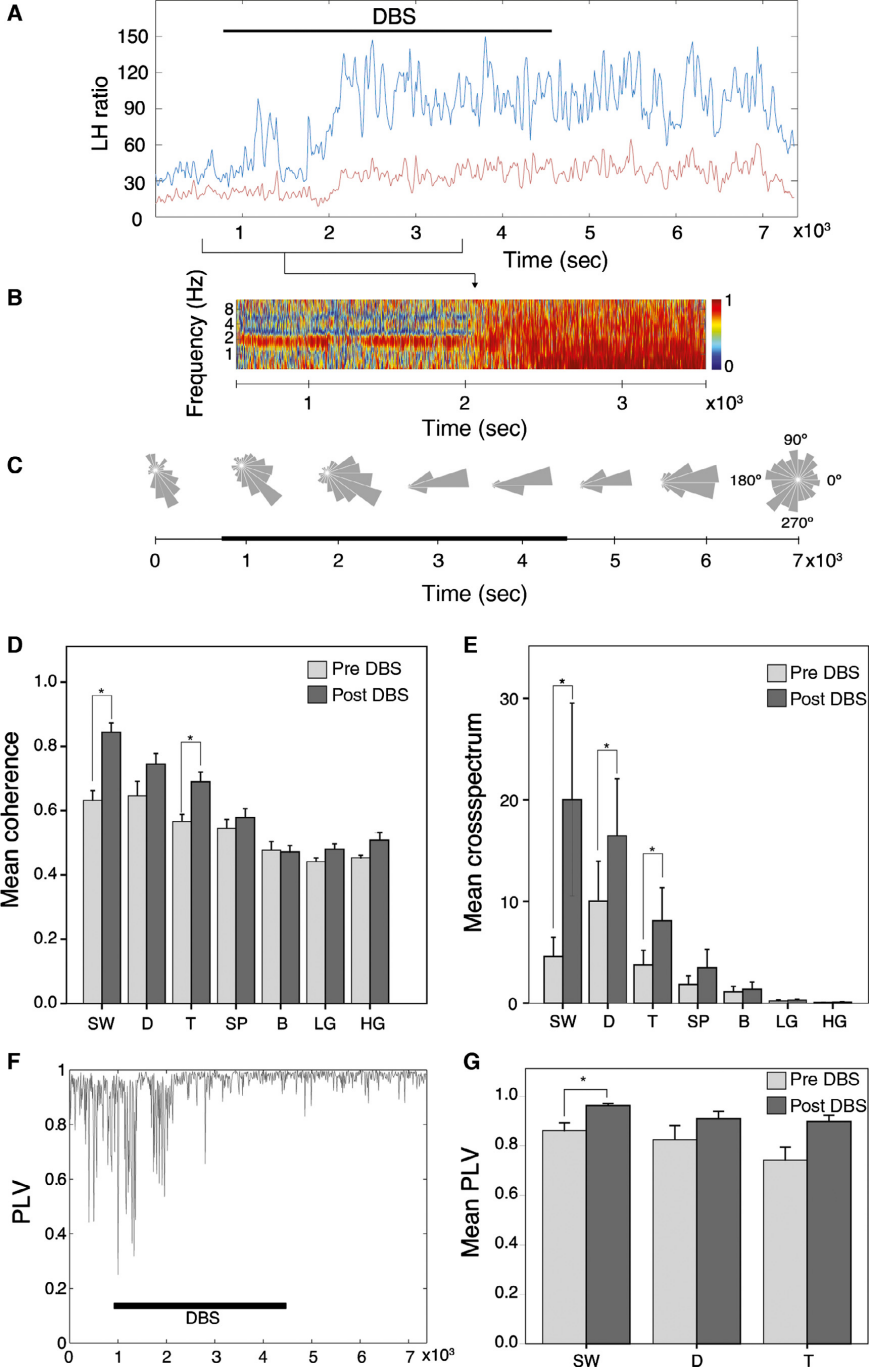
The SW generated after the onset of DBS (indicated by the horizontal line) persisted after the offset of DBS. (A) Hippocampal LH ratio (blue) increased significantly following DBS whereas, with a similar temporal pattern, LH ratio of the BLA (red) did not change significantly. (B) Color‐coded coherogram between hippocampus and BLA before and after the DBS. Note the increase below 1.5 Hz and theta frequencies after the DBS. (C) Phase diagrams along the full recording, demonstrating phase locking under the effect of DBS. Target phase was at the trough of the SW cycle. Basal and post‐DBS effect epochs showed a partial loss of phase locking. Statistical data showing a synchronized state between the hippocampus and BLA following DBS. (D) Both LFPs show a significant correlation in SW, delta, and theta bands with the application of the DBS. (E) The spectral coherence values indicate differences in SW, delta, and theta bands between basal and DBS conditions. (F) Representative case of the temporal evolution (10‐sec window) of the phase‐locking value in the SW band. With a delay after the onset of the DBS, the phase of the oscillations become highly coupled, with values close to 1. This coupling remains after the cessation of DBS. (G) Statistical data showing that the phase locking occurs within the SW band but not in delta or theta frequencies. Results expressed as mean ± SEM (*P <* *0.05, Wilcoxon test). SW, slow oscillations; D, delta band; T, theta band; S, spindle band; LG, low‐gamma band; HG, high‐gamma band; FO, fast oscillations; DBS, Deep brain stimulation.

We also assessed the distribution of the power spectra using the low/high (L/H) ratio, that is, the power ratio between the 0.4–4 Hz and higher frequencies (20–55 Hz). A high L/H ratio characterizes synchronized states, whereas a low L/H ratio indicates desynchronization (Li et al. 2009). As shown in Figure 3A, DBS increased the mean L/H ratio in the hippocampus from 54.60 ± 20.61 to 105.02 ± 24.88 after DBS (*P *<* *0.05; Wilcoxon test). In the BLA the change from 47.75 ± 13.18 to 68.97 ± 6.63 was not statistically significant (*P *=* *0.075; Wilcoxon test).

### Effects of DBS on coupling between hippocampal and BLA

A representative wavelet coherogram showed a clear change after DBS application with higher values at SW frequencies (Fig. 3B). A typical example of phase‐locking phenomenon was shown in Figure 3C. A narrow shape of the phase difference distribution between both areas following DBS indicates a phase coupling profile. To compare oscillatory patterns between hippocampal and BLA signals, the wavelet coherence (Fig. 3D) and the wavelet cross‐spectrum (Fig. 3E) of the signals in 30 10‐sec windows were calculated. DBS produced increases (Wilcoxon test) of mean cross‐spectrum values in SW (from 4.59 ± 1.89 to 20.03 ± 9.49; *P *<* *0.05), delta (from 10.03 ± 3.9 to 16.47 ± 5.62; *P *<* *0.05), and theta (from 3.77 ± 1.43 to 8.11 ± 3.24; *P *<* *0.05) frequencies. Similarly, the coherence values between hippocampus and BLA for SW changed from 0.63 ± 0.03 in the baseline to 0.84 ± 0.03 after DBS (*P *<* *0.05; Wilcoxon test). In the theta band, the coherence also increased from 0.57 ± 0.02 to 0.69 ± 0.03 (*P *<* *0.05, Wilcoxon test).

The frequency bands that showed a change in the coupling between hippocampus and BLA were further analyzed for phase‐locking calculation. As a rule, basal conditions exhibit high PLV, above 0.7, which became better phase‐locked after DBS (Fig. 3F). In the conditions of this study, only SW exhibited a significant increase in the PLV (Fig. 3G), from 0.86 ± 0.03 to 0.96 ± 0.01 (*P *<* *0.05, Wilcoxon test). However, although both delta (0.82 ± 0.06 vs. 0.91 ± 0.03, *P *=* *0.075, Wilcoxon test) and theta (0.74 ± 0.05 vs. 0.90 ± 0.03; *P *=* *0.075, Wilcoxon test) bands showed a similar tendency to increase the phase locking after DBS, these changes did not reach statistical significance.

### Effects of DBS on phase‐amplitude modulation

Our study also included the analysis of the phase‐amplitude modulation between different frequency bands for each LFP site. It is well‐known that SW can propagate through long distances and implicate a large population of neurons. In contrast, higher frequency bands represent a more confined transfer of information. Therefore, the MI of phase‐amplitude coupling is indicative of the existence of local neuronal activity coupled to the network of structures associated with SW.

**Figure 4:**
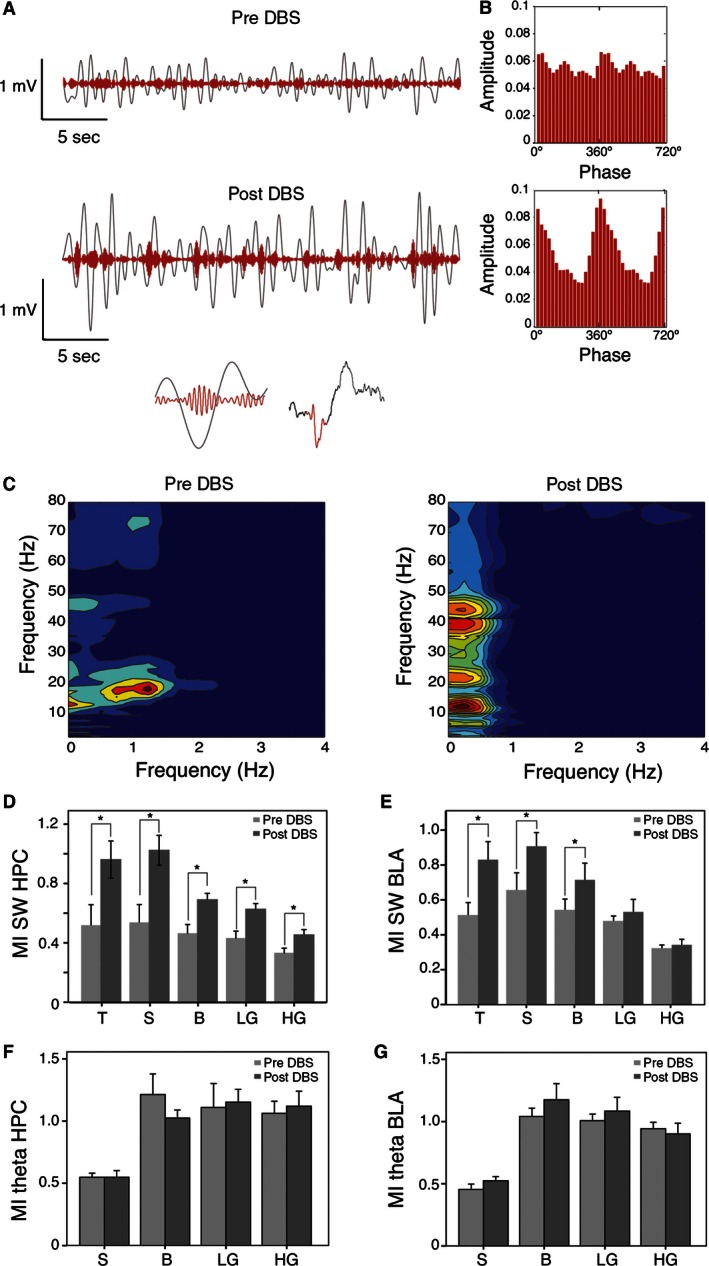
The slow oscillations under DBS effect are coupled with higher frequencies. (A) Representative example showing the coupling between the filtered hippocampal signal for slow oscillations (blue) and spindles (red). Note that after the DBS, the amplitude of the spindles is maximal around the valley of the slow wave. (B) Diagrams showing the comodulation index for the epochs represented in (A). The comodulogram corroborates the coupling between the amplitude of the spindles with the 0°/360° (up‐state) of the slow oscillation. (C) Representation of the frequency‐amplitude modulation for frequencies in the 0–4 Hz versus 0–80 Hz in the hippocampus. In the post‐DBS period, the slow oscillations (but not the delta band) comodulates with a wide spectrum of frequencies in the theta, spindles, beta, and gamma bands. Statistical data show the changes in phase‐amplitude modulation index between SW in the hippocampus (D) and BLA (E), and theta in the hippocampus (F) and BLA (G) with higher frequencies (mean ± SEM; *P <* *0.05, Wilcoxon test). HPC, hippocampus; BLA, basolateral amygdala; DBS, deep brain stimulation; T, theta band; S, spindle band; B, beta band; LG, low‐gamma band; HG, high‐gamma band.

As shown in Figure 4A (Top), spindle amplitude is coupled to SW phase under basal (pre‐DBS) conditions. After DBS, this phase‐amplitude coupling was heightened (Fig. 4A, bottom). This increase in cross‐frequency coupling after DBS is more patent in the phase‐amplitude diagrams (Fig. 4B). Our results showed coupling between the phase of the SW rhythm and the amplitude of broadband activity (Fig. 4C). Thus, in the hippocampus (Fig. 4D), DBS induced significant increases of MI between the phase of SW and the amplitude of theta activity, spindles, beta, slow gamma, and fast gamma. Similar results were found in the BLA (Fig. 4E), where DBS induced significant increases of MI between the phase of SW and the amplitude of theta activity, spindle waves, and beta bands. All comparisons described were made using the Wilcoxon test and illustrated in Table 1. In contrast, the MI between the phase of theta band and the amplitude of the other higher frequency bands was not altered by DBS in hippocampus (Fig. 4F) and BLA (Fig. 4G).

**Table 1:** Changes in MI between the phase of SW and the amplitude of different frequency bands for hippocampus and basolateral amygdala

	Theta		Spindles		Beta		Slow gamma		Fast gamma	
	Pre‐DBS	Post‐DBS	Pre‐DBS	Post‐DBS	Pre‐DBS	Post‐DBS	Pre‐DBS	Post‐DBS	Pre‐DBS	Post‐DBS
Hippocampus	5.18 ± 1.39	9.61 ± 1.26^a^	5.37 ± 0.12	10.24 ± 1.01^a^	4.65 ± 0.59	6.90 ± 0.43^a^	4.31 ± 0.48	6.26 ± 0.38^a^	3.32 ± 0.32	4.54 ± 0.35^a^
Basolateral amygdala	5.10 ± 0.74	8.30 ± 1.04^a^	6.53 ± 1.02	9.07 ± 0.79^a^	5.40 ± 0.65	7.14 ± 0.97^a^	4.77 ± 0.30	5.31 ± 0.72	3.20 ± 0.21	3.42 ± 0.32

Values (×10^−3^) are expressed as mean ± SEM. DBS, deep brain stimulation; MI, modulation index; SW, slow wave.

a *P *<* *0.05, Wilcoxon test.

### Effects of DBS on mutual information

**Figure 5:**
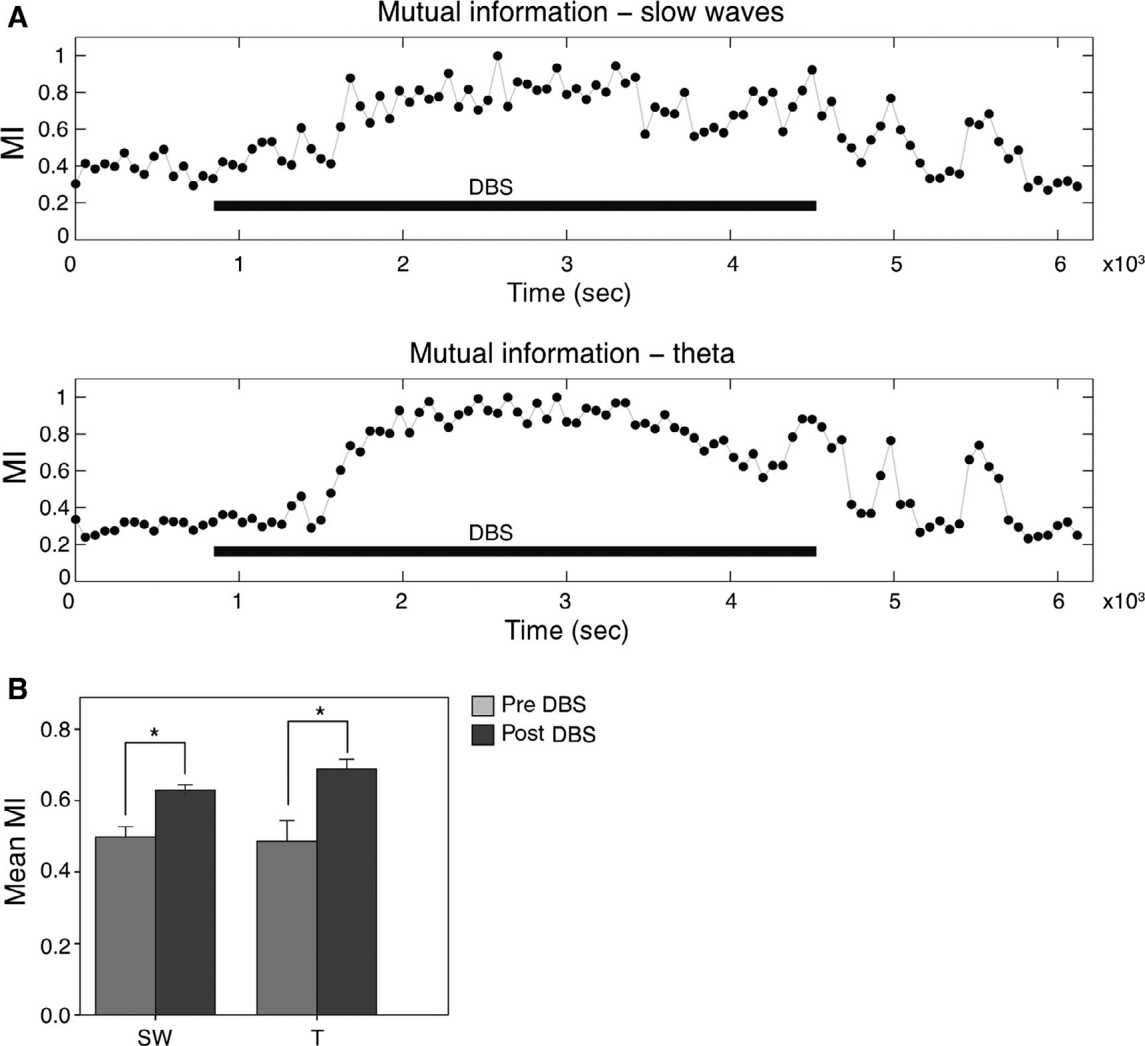
The hippocampal‐amygdalar complex increases the communication flow as an effect of the DBS of the infralimbic cortex. (A) Representative case showing the temporal evolution of the mutual information between the hippocampus and the basolateral amygdala (60 sec‐window). With a delay after the onset of the DBS, the exchange of information increases within the slow oscillation and theta bands. (B) Statistical data showing the increase in mutual information in the slow waves and theta bands after the DBS. Values expressed as mean ± SEM (*P <* *0.05, Wilcoxon test). DBS, deep brain stimulation; SW, slow wave oscillation; T, theta band.

To investigate the information processing in the prefrontal‐hippocampus‐amygdala network, the mutual information was calculated by considering only the dominant SW and theta oscillations. Consequently, this parameter estimates the transfer of information between the hippocampus and BLA, before and after DBS. As shown in Figure 5, the comparisons (Wilcoxon test) revealed that 1 h DBS increased mutual information between hippocampus and amygdala in the SW (from 0.50 ± 0.03 to 0.63 ± 0.02; *P *<* *0.05) and theta (from 0.49 ± 0.06 to 0.69 ± 0.03; *P *<* *0.05) frequencies.

## Discussion

The first main result of this study is that 1 h IL DBS induced an increase in the power of SW (<1.5 Hz) and theta (3–12 Hz) frequencies both in the hippocampus and the BLA in the urethane‐anesthetized rat. Previous work has also showed that IL DBS also increased slow oscillatory activities within the stimulated area (Etiévant et al. 2015), which is thought to reflect synchronous changes in membrane potential caused by recurrent cortical activity (Sanchez‐Vives and McCormick 2000; Shu et al. 2003). Reduced slow oscillatory activity has been reported in two different animal models of depression (Voget et al. 2015; Zheng and Zhang 2015), and clinical studies have shown decreased SW activity in depression that is restored after effective antidepressant treatments (Thase et al. 1997; Duncan et al. 2013). Accordingly, reduced sleep SW activity (ranging from 1 to 4 Hz) has been postulated as biomarker of MDD (Duncan and Zarate 2013). Here, we extend this assertion to lower frequencies (<1.5 Hz), which might represent a useful biomarker of depressive states. Furthermore, the positive correlation between serum BDNF level and SW activity suggests a close relationship between SW and synaptic plasticity (Duncan and Zarate 2013). Both SW and theta bands have been associated with the formation of long‐term memory (Hasselmo 2005). Hence, increased activity and coherence in the theta band might result in greater command on information processing within this circuit, thus resulting in improved cognitive functions. Furthermore, the increased coherence in the theta band between hippocampus and BLA could represent a homeostatic mechanism to correct the impaired control of emotional stimuli, which is characteristic of depressive states.

On the other hand, SW and delta oscillations appear in quiet wakefulness and non‐REM sleep phases. Urethane anesthesia emulates sleep EEG patterns, alternating SW and delta epochs with periods of predominance of theta oscillations (Buzsáki 2002). Taken together, DBS‐induced changes in oscillatory patterns may be related to the improvement of symptoms associated with depressive states. In general, DBS produced a greater synchronous activity at low frequencies in the hippocampus when compared with the BLA, as measured by increased high L/H ratio. The lack of effect of L/H ratio in BLA was likely due to a relative, parallel increase in the power of fast frequencies.

In addition, our results showed that IL DBS causes a precise coupling in different frequency bands between both brain structures. This was reflected first, by an enhanced coherence and cross‐spectrum values between hippocampus and BLA in SW and theta bands; second, by an increased phase‐locking value in SW rhythm between hippocampus and BLA; and third, DBS also elevates mutual information, which indicates an increased amount of information shared between hippocampus and BLA. This increase was also observed in SW and theta frequencies. All these findings reveal a facilitation of the communication in the hippocampus‐BLA network. Furthermore, phase‐amplitude coupling between different frequency bands for each LFP site also revealed increased modulation between the SW phase and the amplitude of faster bands both in the hippocampus and BLA. SW can propagate across long distances, whereas faster bands have more limited spatial influence. Therefore, the enhanced modulation index between SW and faster frequencies in hippocampus and BLA suggests the existence of a local neuronal activity coupled to a network of different brain structures functionally connected with SW activity.

The mPFC is connected to different brain regions that mediate the multiple conditions of depressive symptomatology such as hippocampus and BLA (Koenigs and Grafman 2009). Projections from mPFC to hippocampus are not direct, but travel mainly through the nucleus reuniens of the thalamus (Hoover and Vertes 2012). On the other hand, the amygdala is largely interconnected with the hippocampus and the mPFC (McDonald et al. 1996; Petrovich et al. 1996). In particular, the IL subregion of the mPFC innervates preferentially GABAergic rich areas of the amygdala (Pinard et al. 2012), known as the intercalated cells that increase their activity as a result of electrical stimulation of IL and exert an inhibitory influence over the amygdalar output (Amir et al. 2011). Thus, it is likely that DBS produces oscillatory changes in mPFC that can be conveyed to the hippocampus and BLA. Given the cellular connections between these brain structures and their functional implications, it can be argued that IL DBS would favor hippocampus‐dependent cognitive processes while reducing amygdala‐dependent emotional and anxiety‐like behaviors.

A number of potential limitations of the study should be noted. An important issue to be taken into account is the use of anesthesia. Although it is thought that anesthetics like urethane can preserve many physiological features, the neuronal synchrony can be regulated differently in the awake and anesthetized animals (Hara and Harris 2002). Thus, the possibility that the oscillatory changes observed might not reflect precisely those in the awake rats cannot be ruled out completely. Another concern is that the study has been carried out in naïve rats and not in an animal model of the illness. However, it is conceivable that the IL DBS changes observed are similar to those produced in depressed individuals at the circuit level (McCracken and Grace 2007). Furthermore, the changes observed take place shortly after DBS offset and additional work is needed to determine the effects after longer periods of stimulation. Finally, although the coupling activity and subsequent transfer of information observed between hippocampus and BLA are clear‐cut, further research is needed to elucidate the direction of this conveyance and the contribution of each region to these effects.

In conclusion, the increases in SW band synchronization in hippocampus and BLA after DBS suggest that these changes may be important for the improvement of depressive behavior. In addition, the augmentation in theta synchrony might contribute to improvement in emotional and cognitive processes. Interestingly, SW activity is also present in brainstem serotonergic and noradrenergic nuclei (Schweimer et al. 2011; Eschenko et al. 2012), and prefrontal 5‐HT is known to increase the frequency and amplitude of SW in the mPFC (Puig and Gulledge 2011). Thus, the increased prefrontal release of these monoamines in the mPFC that we have found in previous work (Jiménez‐Sánchez et al. 2016) would also be compatible with the enhancement of oscillatory activity in the SW band seen in this study. However, further studies should be undertaken in an animal model of depression to validate the present findings.

## Conflict of Interest

The authors declare no financial conflict of interest.

## Supporting information




**Figure S1.** Changes in power spectra in the hippocampus after 1 h IL DBS.
**Figure S2.** Changes in power spectra in the BLA after 1 h IL DBS.
