# Supplementary material for: Characterization of oscillatory changes in hippocampus and amygdala after deep brain stimulation of the infralimbic prefrontal cortex
Source: Physiol Rep. 2016 Jul 22;4(14):e12854. doi: 10.14814/phy2.12854 (PMC4962070; doi:10.14814/phy2.12854)
Supplement: Supplementary file 1 — Figure S1. Changes in power spectra in the hippocampus after 1 h IL DBS. Figure S2. Changes in power spectra in the BLA after 1 h IL DBS. [file PHY2-4-e12854-s001.docx]

**Characterization of oscillatory changes in hippocampus and amygdala after deep brain stimulation of the infralimbic prefrontal cortex**

Ana Cervera-Ferri^1,+^, Vicent Teruel-Martí^1,+^, Moises Barceló-Molina^1,2^, Joana Martínez-Ricós^1^, Aina Luque-García^1,2^, Sergio Martínez-Bellver^1,3^ and Albert Adell^4,*^

^1^Neuronal Circuits Laboratory, Department of Human Anatomy and Embriology, Faculty of Medicine and Odontology, University of Valencia, 46010 Valencia, Spain.

^2^Instituto de Investigación Sanitaria La Fe, 46026 Valencia, Spain.

^3^Department of Cell Biology and Parasitology, Faculty of Medicine and Odontology, University of Valencia, 46010 Valencia, Spain.

^4^Institute of Biomedicine and Biotechnology of Cantabria, IBBTEC (CSIC, University of Cantabria), 39011 Santander, Spain.

***Supplementary Information***


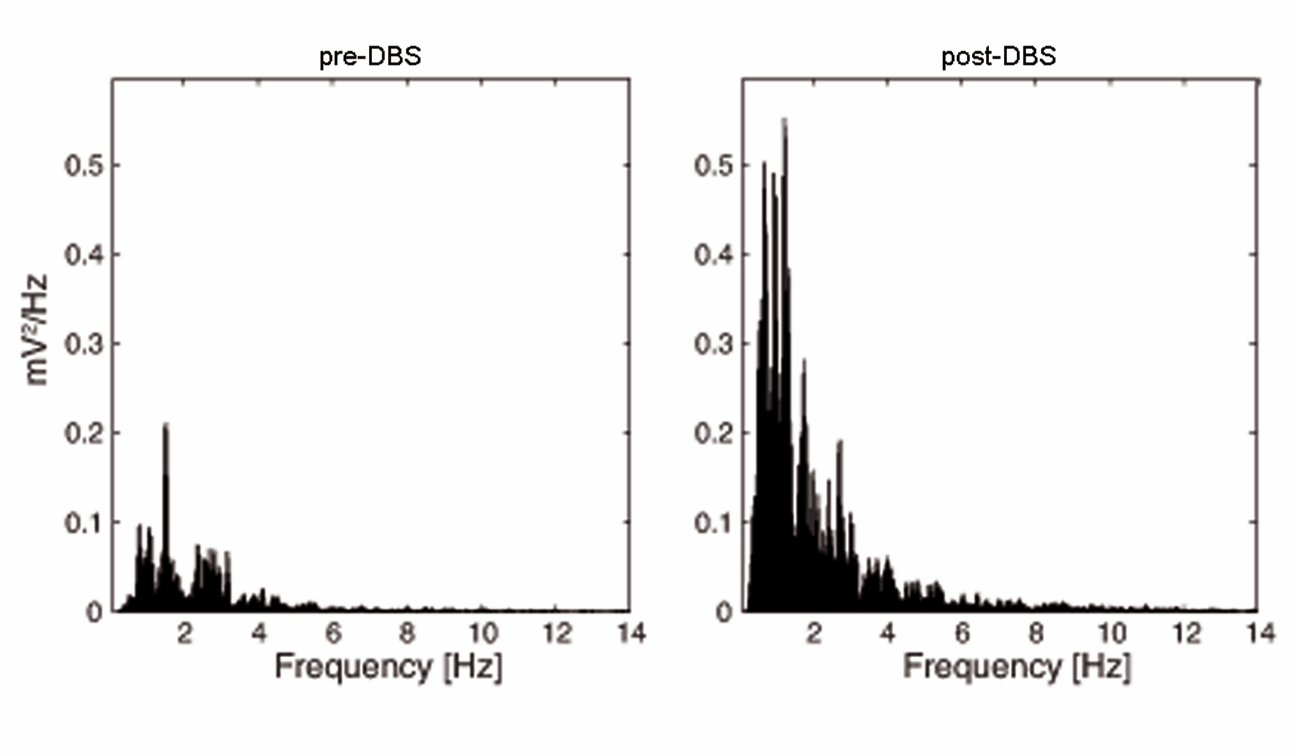


**Figure S1.** Changes in power spectra in the hippocampus after 1 h IL DBS.


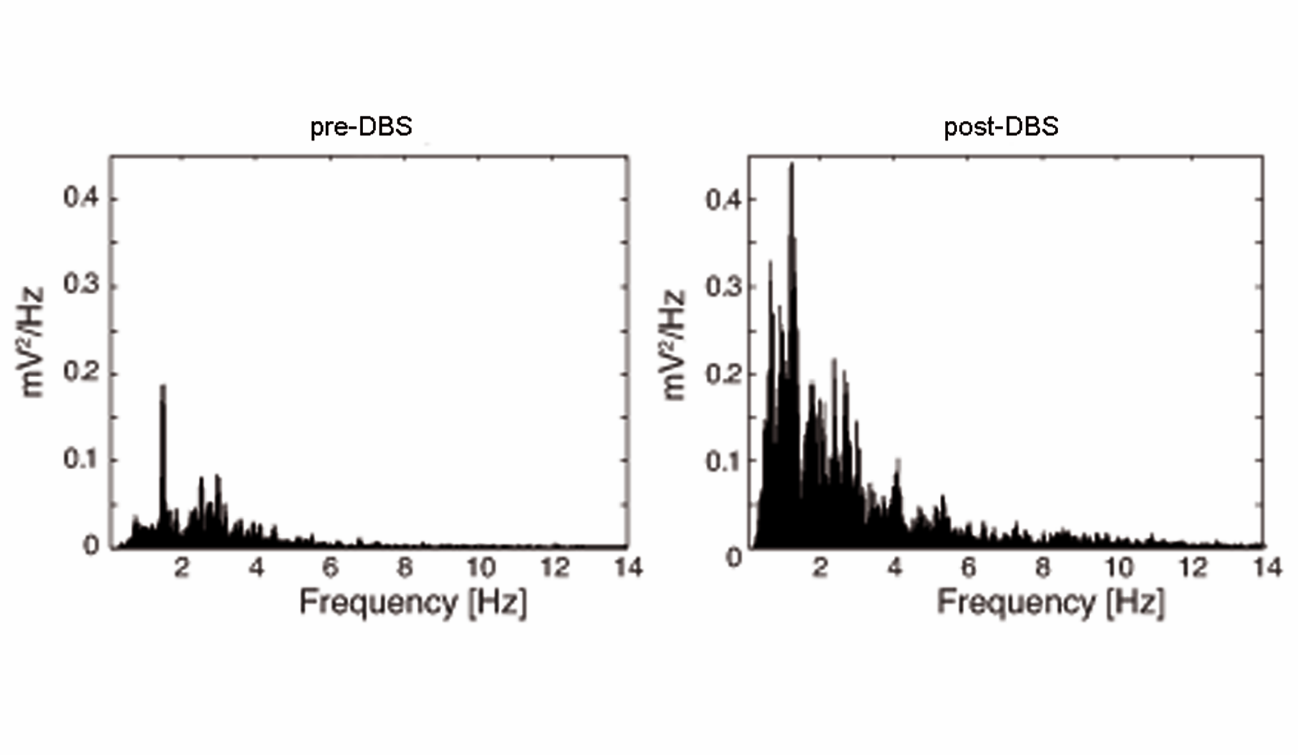


**Figure S2.** Changes in power spectra in the BLA after 1 h IL DBS.
